# Healthcare utilization and preventive care among social housing residents compared to the general population during the COVID-19 pandemic in Ontario, Canada: a population-based cohort study

**DOI:** 10.3389/fpubh.2025.1729767

**Published:** 2026-01-06

**Authors:** Gina Agarwal, Homa Keshavarz, Ricardo Angeles, Melissa Pirrie, Francine Marzanek, Francis Nguyen, Jasdeep Brar, J. Michael Paterson

**Affiliations:** 1 Department of Family Medicine, McMaster University, Hamilton, ON, Canada; 2 ICES, Toronto, ON, Canada; 3 Institute of Health Policy, Management and Evaluation, University of Toronto, Toronto, ON, Canada

**Keywords:** administrative health data, cancer screening, COVID-19, health equity, influenza vaccination, preventive health services, social housing

## Abstract

**Introduction:**

To examine disparities in influenza vaccination and screening for breast, cervical, and colorectal cancers among adults living in social housing compared to the general population of Ontario, Canada, before and during the COVID-19 pandemic.

**Methods:**

A population-based cohort study was conducted using linked administrative health data from Ontario, Canada. We studied individuals aged 18 and older who were alive on January 1, 2020. Social housing sites were identified using the 2023 cycle of the Social Housing of Ontario Registry. The complement cohort comprised adults not residing in social housing. Receipt of influenza vaccination and screening for cervical (Pap test), breast (mammography), and colorectal [fecal immunochemical test (FIT)/fecal occult blood test (FOBT)/sigmoidoscopy/colonoscopy] cancers were analyzed using data from the Ontario Breast Screening Program (OBSP), Ontario Cancer Registry (OCR), Ontario Health Insurance Plan (OHIP) Database, and Primary Care Population Database (PCPOP). Age-standardized rates and rate differences between the social housing population and complement cohort were compared for the pre-COVID-19 (2018 and 2019) and COVID-19 (2020 and 2021) periods.

**Results:**

The social housing cohort included 297,644 individuals, while the complement cohort had 11,386,078 individuals. The social housing cohort had higher proportions of older adults (≥60 years) and females. Age- and sex-standardized disparities in influenza vaccination (≥1 dose in 2-year period) between social housing residents and the complement cohort widened during the COVID-19 pandemic from −0.30 to −1.84%, with the largest gaps observed among adults aged 80 and older. While age- and sex-standardized disparities in breast and cervical cancer screening narrowed, they remained significant. In contrast, the age- and sex-standardized disparity in colorectal cancer screening increased from −7.42 to −9.69%, particularly among males and older adults aged 60–74.

**Discussion:**

Disparities in healthcare utilization and preventive care between social housing residents and the complement cohort persisted or widened during the COVID-19 pandemic, most notably for influenza vaccination and colorectal cancer screening. Narrowing of some screening disparities was primarily attributed to overall declines in screening rates rather than improved access. These findings emphasize the need for targeted, equity-focused public health strategies to improve access to preventive healthcare services for socially and economically disadvantaged populations.

## Introduction

In the aftermath of the COVID-19 pandemic, understanding its continued impact on healthcare access and utilization represents essential information for healthcare system decision-makers tasked with mitigating these impacts and preparing for future emergencies. Healthcare access and utilization were significantly disrupted during the pandemic, disproportionately affecting population groups (1–3). Studies have specifically documented notable declines in preventive services use and access, particularly among vulnerable communities (4). Decreased preventative service use can lead to missed diagnoses, poorer long-term health outcomes, and difficulty in restoring routine population-level screening rates to pre-pandemic levels due to backlogs and reduced patient uptake.

Social housing programs, a critical subset of affordable housing, provide subsidized rental units for low-income individuals (5). In Ontario, Canada, approximately 363,819 people aged 5 years and older resided in social housing between January 1, 2020, and December 31, 2021 (6). This population faces significant social and health vulnerabilities, including a higher burden of chronic diseases and poorer overall health compared to the general population (7, 8). Adults aged 40 and older in social housing report substantially higher rates of cardiovascular disease and diabetes (8–10). These health disparities are compounded by limited access to and uptake of preventive healthcare services such as cancer screenings and vaccinations, which may have been exacerbated during the pandemic (8, 11). Chronic conditions are associated with reduced quality of life and increased healthcare costs, making them a public health priority. Primary care guidelines in Ontario recognize the importance of preventive care and, during the study period, recommended regular breast cancer screening for individuals aged 50–69, cervical cancer screening for those aged 21–69, colorectal cancer screening for adults aged 50–74, and annual influenza vaccination across all age groups (12–15). Historically, influenza vaccinations have been primarily delivered in primary care settings, while cancer screening services are provided across multiple settings: mammograms are performed in community radiology centers, Pap tests are offered in primary care settings, and fecal immunochemical test (FIT) tests are mailed directly to patients' homes. These primary care preventive measures are important for early disease detection, prevention, and management, contributing to improving population health outcomes.

Although understanding the primary care preventive care practices of social housing residents is critical, research on this topic remains limited. Understanding how the COVID-19 pandemic affected healthcare utilization in this population is crucial for shaping responsive policies and developing targeted strategies to improve access to preventive services. It also provides a foundation for planning health systems recovery efforts in screening and diagnostic services. This study explores the impact of the COVID-19 pandemic on healthcare utilization and preventive care, specifically cancer screening and influenza vaccination, in social housing residents compared to the general population in Ontario, Canada.

## Materials and methods

### Study design and data sources

A retrospective population-based cohort study was conducted using administrative healthcare data from Ontario, Canada's most populous province. The data were securely linked using unique encoded identifiers and analyzed at ICES, a not-for-profit research institute funded by the Ontario Ministry of Health (http://www.ices.on.ca/).

The Ontario Health Insurance Plan (OHIP) Registered Persons Database (RPDB) was used to determine health insurance status, demographic characteristics, and residential postal code. Physician service claims were obtained from OHIP Claims History Database. Cancer history was collected from the Ontario Cancer Registry (OCR). Breast, cervical, and colorectal cancer screening and influenza vaccination were identified from a combination of records in the Ontario Breast Screening Program (OBSP), Primary Care Population Database (PCPOP), and OHIP Claims History Database. These databases were securely linked at the individual level and analyzed in an anonymous, coded form at ICES under Section 45 of Ontario's Personal Health Information Protection Act.

### Study population and setting

The study included two cohorts of Ontario adults aged 18 and older who were alive on January 1, 2020. The first cohort comprised individuals residing in social housing buildings, which were sites identified in the 2023 cycle of the Social Housing of Ontario Registry (16). The second, or complement, cohort consisted of individuals in the general population who were not residing in social housing. Detailed cohort derivation methods have been described previously (6, 8, 11).

### Study measures

The study measures were influenza vaccination and screening for cervical, breast, and colorectal cancer. Eligibility for the “Pre” COVID-19 period was determined as of January 1, 2018, and for the “During” COVID-19 period was as of January 1, 2020. All study measures except colorectal cancer screening were based on administrative data between January 1, 2018, and December 31, 2019, for the “Pre” period and between January 1, 2020, and December 31, 2021, for the “During” period. Individuals were categorized as having received at least one influenza vaccination if they had an OHIP billing code for influenza vaccination in each 2-year period (17). Cervical cancer screening (Pap test) eligibility included females aged 21–69 years, according to the RPDB, with no history of cervical cancer or hysterectomy based on OHIP billing codes and OCR entries. Pap test screening was identified using specific OHIP billing codes. Eligibility for breast cancer screening (mammography) included females aged 50–69 years in the RPDB, with no history of breast cancer or mastectomy, based on OHIP billing records and OCR entries. Mammography screenings conducted as part of the OBSP, as well as those performed outside the program, were included (18). Colorectal cancer screening eligibility included individuals aged 52–74 years, excluding high-risk individuals with a history of colorectal cancer or inflammatory bowel disease. Screening status was determined using the PCPOP database, a population-level ICES database that includes all Ontario residents eligible for OHIP and provides information on health services use, including cancer screening status. Individuals were considered up-to-date if they had at least one fecal immunochemical test (FIT) or fecal occult blood test (FOBT) in the previous 2 years, or a flexible sigmoidoscopy or colonoscopy in the past 10 years (19).

### Statistical analysis

The characteristics of the cohorts were summarized using descriptive statistics and grouped according to public health unit regions used by a provincial surveillance program at the time of the data (2020) (20). Influenza vaccination and breast, cervical, and colorectal cancer screening rates were estimated for the social housing and complement cohorts during the “Pre-COVID-19” (2018–2019) and “During COVID-19” (2020–2021) time periods. Age- and sex-standardized rates for the social housing population were calculated using the direct method of standardization (21), with the age and sex population structure of the complement cohort serving as the reference. The rates were estimated overall and across age and sex groups, where applicable. Differences in vaccination and screening rates between the two cohorts, and their 95% confidence intervals (CIs) during “Pre-COVID-19” and “During COVID-19” time periods, were calculated using the method recommended by Altman et al. (22) and presented as percentages.

## Results

There were 297,644 individuals identified as residing in social housing and constituted the social housing cohort, while 11,386,078 individuals not residing in social housing comprised the complement cohort.

Compared to the complement cohort, the social housing cohort had a lower percentage of individuals in the younger age groups (18–39 years: 35.70 vs. 37.03% and 40–59 years: 28.71 vs. 34.05%), and a higher percentage in the older age groups (60–79 years: 26.71 vs. 24.19% and 80+ years: 8.88 vs. 4.73%; Table 1). The percentage of males was lower in the social housing cohort than in the complement cohort (41.44 vs. 49.08%), while the percentage of females was significantly higher (58.56 vs. 50.92%) (see sex distribution across age categories in Table 2).

**Table 1:** Demographics in the social housing and complement cohorts as of January 1st, 2020.

**Demographics**	**Social housing cohort (*n* = 297,644) *n* (%)**	**Complement cohort (*n* = 11,386,078)*n* (%)**
**Age group (years)**		
18–39	106,269 (35.70)	4,216,343 (37.03)
40–59	85,445 (28.71)	3,876,523 (34.05)
60–79	79,502 (26.71)	2,755,004 (24.19)
80+	26,428 (8.88)	538,208 (4.73)
**Sex**		
Male	123,329 (41.44)	5,588,120 (49.08)
Female	174,315 (58.56)	5,797,958 (50.92)
**Social housing type**		
Government owned public	145,731 (48.96)	–
Other (not-for-profit and co-op)	128,526 (43.18)	–
Not available	23,387 (7.86)	–
**Ontario public health unit region**		
Toronto	112,369 (37.75)	2,229,634(19.58)
Central West	55,679 (18.71)	2,190,317 (19.24)
Central East	44,744 (15.03)	3,424,242 (30.07)
Ottawa	29,917 (10.05)	783,081 (6.88)
South West	23,801 (8.00)	1,168,584 (10.26)
Northern	14,267 (4.79)	648,943 (6.70)
Eastern	12,457 (4.19)	707,446 (6.21)
Not available	4,410 (1.48)	233,831 (2.05)

**Table 2:** Sex distribution across age categories of the social housing and complement cohorts as of January 1st, 2020.

**Age group (years)**	**Overall**, ***n*** **(%)**		**Male**, ***n*** **(%)**		**Female**, ***n*** **(%)**	
	**Social housing cohort (*****n*** = **297,644)**	**Complement cohort (*****n*** = **11,386,075)**	**Social housing cohort (*****n*** = **123,329)**	**Complement cohort (*****n*** = **5,588,120)**	**Social housing cohort (*****n*** = **174,315)**	**Complement cohort (*****n*** = **5,797,958)**
18–39	106,269 (35.70)	4,216,343 (37.03)	48,329 (39.19)	2,119,969 (37.94)	57,940 (33.24)	2,096,374 (36.16)
40–59	85,445 (28.71)	3,876,523 (34.05)	35,413 (28.71)	1,913,405 (34.24)	50,032 (28.70)	1,963,118 (33.86)
60–79	79,502 (26.71)	2,755,004 (24.20)	31,523 (25.56)	1,325,366 (23.72)	47,979 (27.52)	1,429,638 (24.66)
80+	26,428 (8.88)	538,208 (4.73)	8,064 (6.54)	229,380 (4.10)	18,364 (10.53)	308,828 (5.33)

Based on geographical distribution, the social housing cohort had a greater percentage of individuals residing in the Toronto (37.75 vs. 19.58%) and Ottawa (10.05 vs. 6.88%) regions, while a lower percentage resided in the Central East Region (15.03 vs. 30.07%) compared to the complement cohort.

### Influenza vaccination

Across all age groups and both sexes, the social housing cohort had lower rates of receiving at least one dose of influenza vaccination within the respective 2-year time period compared to those in the complement cohort (Table 3). This disparity became more pronounced during the COVID-19 period. The overall age- and sex-standardized difference between the social housing and the complement cohorts increased from −0.30% (95% CI: −0.42 to −0.19%) to −1.84% (95% CI: −1.95 to −1.74%). In the “During COVID-19” period, the social housing cohort had consistently lower sex-standardized vaccination rates across all age categories compared to the complement cohort (range: −1.13 to −3.20%), with the largest difference observed in the 80+ age group (−3.20%, 95% CI: −3.68 to −2.71%). The degree of disparity during COVID-19 was similar for males and females between the social housing and complement cohorts with age-standardized differences of −1.85% (95% CI: −2.00 to 1.69%) for males and −1.83% (95% CI: −1.98 to 1.68%) for females.

**Table 3:** Differences in receipt of at least one dose of influenza vaccination between social housing and complement cohorts in the “Pre” (2018/2019) and “During” (2020/2021) COVID-19 time periods, stratified by age and sex.

**Age group (years)**	**Sex**	**Eligible population**		**At least one influenza vaccination in 2018/2019**		Difference 95% CI Social housing vs. complement cohort (Reference)^*^	**At least one influenza vaccination in 2020/2021**		Difference 95% CI Social housing vs. complement cohort (Reference)^*^
		**Complement cohort (*****n*** = **11,386,075)**	**Social housing cohort (*****n*** = **297,644)**	**Complement cohort** ***n*** **(%)**	**Social housing cohort** ***n*** **(%)**		**Complement cohort** ***n*** **(%)**	**Social housing cohort** ***n*** **(%)**	
18–39	Males	2,119,969	48,329	96,428 (4.55)	1,655 (3.42)	−1.13% (−1.29 to −0.97%)	104,403 (4.92)	1,428 (2.95)	−1.97% (−2.12 to −1.82%)
	Females	2,096,374	57,940	174,443 (8.32)	4,163 (7.19)	−1.13% (−1.34 to −0.92%)	182,435 (8.70)	3,530 (6.09)	−2.61% (−2.81 to −2.41%)
	Overall	4,216,343	106,269	270,871 (6.42)	5,626 (5.29)^**^	−1.13% (−1.26 to −0.99%)	286,838 (6.80)	4,798 (4.51)^**^	−2.29% (−2.41 to −2.16%)
40–59	Males	1,913,405	35,413	157,816 (8.25)	3,113 (8.79)	0.54% (0.24 to 0.84%)	159,322 (8.33)	2,586 (7.30)	−1.03% (−1.30 to −0.76%)
	Females	1,963,118	50,032	195,629 (9.97)	5,279 (10.66)	0.69% (0.42 to 0.96)	199,773 (10.18)	4,469 (8.93)	−1.25% (−1.50 to −1.00%)
	Overall	3,876,523	85,445	353,445 (9.12)	8,273 (9.68)^**^	0.56% (0.36 to 0.76%)	359,095 (9.26)	6,945 (8.13)^**^	−1.13% (−1.32 to −0.95%)
60–79	Males	1,325,366	31,523	251,720 (18.99)	5,764 (18.29)	−0.70% (−1.13 to −0.27%)	239,765 (18.09)	4,934 (15.65)	−2.44% (−2.85 to −2.03%)
	Females	1,429,638	47,974	283,505 (19.83)	9,784 (20.39)	0.56% (0.19 to 0.93%)	272,726 (19.08)	8,498 (17.71)	−1.37% (−1.72 to −1.02%)
	Overall	2,755,004	79,502	535,225 (19.43)	15,407 (19.38)^**^	−0.05% (−0.33 to 0.23%)	512,491 (18.60)	13,294 (16.72)^**^	−1.88% (−2.14 to −1.61%)
80+	Males	229,380	8,064	56,109 (24.46)	1,885 (23.38)	−1.08% (−2.02 to −0.14%)	51,649 (22.52)	1,476 (18.30)	−4.22% (−5.08 to −3.36%)
	Females	308,828	18,364	75,360 (24.40)	4,191 (22.82)	−1.58% (−2.21 to −0.95%)	68,174 (22.08)	3,604 (19.63)	−2.45% (−3.04 to −1.86%)
	Overall	538,208	26,428	131,469 (24.43)	6,094 (23.06)^**^	−1.37% (−1.89 to −0.85%)	119,823 (22.26)	5,038 (19.06)^**^	−3.20% (−3.68 to −2.71%)
Overall	Males (age standardized)	5,588,120	123,329	562,073 (10.06)	11,846 (9.61)	−0.45% (−0.62 to −0.29%)	555,139 (9.93)	9,971 (8.08)	−1.85% (−2.00 to −1.69%)
	Females (age standardized)	5,797,958	174,315	728,937 (12.57)	21,641 (12.41)	−0.16% (−0.31 to −0.00%)	723,105 (12.47)	18,548 (10.64)	−1.83% (−1.98 to −1.68%)
	Overall (age and sex standardized)	11,386,078	297,644	1,291,010 (11.34)	32,848 (11.04)	−0.30% (−0.42 to −0.19%)	1,278,247 (11.23)	27,937 (9.39)	−1.84% (−1.95 to −1.74%)

^*^A negative sign implies a lower proportion of individuals in social housing compared to the complement cohort.

^**^Age- and sex-standardized rates for social housing using the complement cohort as reference.

### Breast cancer screening

Across both time periods, individuals in social housing had consistently lower rates of breast cancer screening (mammography) compared to those in the complement cohort (Table 4). Among females aged 50–69 years in the “Pre-COVID-19” period (2018–2019), the age-standardized mammography rate for breast cancer screening was 46.37% in the social housing cohort and 56.95% in the complement cohort, representing a difference of −10.58% (95% CI: −11.02 to −10.14%). This difference was similarly reflected across the 50–59 and 60–69 age groups, at −10.23 and −11.0%, respectively.

**Table 4:** Breast and cervical cancer screening (mammography and Pap test) among females in the social housing and complement cohorts in the “Pre” (2018/2019) and “During” (2020/2021) COVID-19 time periods, stratified by age.

**Cancer screening**	**Age group (years)**	**Eligible population**		**2018/2019 incidence rate**			**2020/2021 incidence rate**		
		**Complement cohort** ***n***	**Social housing cohort** ***n***	**Complement cohort*****n*** **(%)**	Social housing^**^n **(%)**	**Difference 95% CISocial housing vs. complement cohort (Reference)** ^*^	**Complement cohort** ***n*** **(%)**	Social housing^**^n **(%)**	**Difference 95% CI Social housing vs. complement cohort (Reference)** ^*^
Number of unique individuals with mammography	50–59	992,082	25,089	528,604 (53.28)	10,802 (43.05)	−10.23% (−10.85 to −9.61%)	213,576 (21.53)	4,353 (17.35)	−4.18% (−4.66 to −3.70%)
	60–69	848,898	24,707	519,901 (61.24)	12,414 (50.24)	−11.00% (−11.63 to −10.37%)	189,001 (22.26)	4,524 (18.31)	−3.95% (−4.44 to −3.46%)
	All ages	1,840,980	49,796	1,048,505 (56.95)	23,090 (46.37)	−10.58% (−11.02 to −10.14%)	402,577 (21.87)	8,860 (17.79)	−4.08% (−4.42 to −3.74%)
Number of unique individuals with a Pap test	21–39	1,842,638	49,697	700,341 (38.01)	16,806 (33.82)	−4.19% (−4.61 to −3.77%)	596,017 (32.35)	14,446 (29.07)	−3.28% (−3.68 to −2.88%)
	40–59	1,935,662	49,250	825,359 (42.64)	17,666 (35.87)	−6.77% (−7.20 to −6.34%)	602,703 (31.14)	12,992 (26.36)	−4.78% (−5.17 to −4.39%)
	60–69	866,020	25,130	318,977 (36.83)	7,089 (28.21)	−8.62% (−9.19 to −8.05%)	201,896 (23.31)	4,574 (18.20)	−5.11% (−5.60 to −4.62%)
	All ages	4,644,320	124,077	1,844,677 (39.72)	41,723 (33.63)	−6.09% (−6.36 to −5.83%)	1,400,616 (30.16)	32,162 (25.92)	−4.24% (−4.49 to −3.99%)

^*^A negative sign implies a lower proportion of individuals in social housing compared to the complement cohort.

^**^Incidence rates for social housing cohort have been standardized using the complement cohort age distribution as the reference.

During the COVID-19 period (2020 to 2021), age-standardized mammography rates declined in both cohorts. The decline was larger in the complement cohort, falling from 56.95 to 21.87%, compared with a decline from 46.37 to 17.79% among the social housing cohort, representing a narrowed disparity of −4.08% (95% CI: −4.42 to −3.74%). Similar patterns were observed across the 50–59 and 60–69 age groups, with differences of −4.18 and −3.95%, respectively.

### Cervical cancer screening

Cervical cancer screening (Pap test) showed persistent disparities between the social housing and complement cohorts, with consistently lower rates observed in the social housing cohort across both time periods and all age groups (Table 4). In the “Pre-COVID-19” period (2018–2019), the overall age-standardized difference in Pap test was −6.09% (95% CI: −6.36 to −5.83%). In “During COVID-19” period (2020–2021), rates decreased in both cohorts, from 39.72 to 30.16% in the complement cohort and from 33.63 to 25.92% in social housing cohort, resulting in a narrowed difference of −4.24% (95% CI: −4.49 to −3.99%). The greatest difference between cohorts was reported in the 60–69 age group, with a difference of −8.62% (95% CI: −9.19 to −8.05%) in the “Pre-COVID-19” period and −5.11%, (95% CI: −5.60 to −4.62%) in the “During COVID-19” period.

### Colorectal cancer screening

Overall, the disparity in age-standardized colorectal cancer screening rates between the social housing and complement cohorts increased from −7.42% (95% CI: −7.72 to −7.12%) in the “Pre-COVID-19” period to −9.69% (95% CI: −10.0 to −9.38%) in the “During COVID-19” period (Table 5). Disparities were greater among males than females in both periods: −10.32 vs. −6.61% in the “Pre-COVID-19” period and −11.52 vs. −7.94% in the “During COVID-19” period. Among females, the largest disparity was observed in those aged 50–59 years (−7.47% Pre-COVID-19 and −8.71% During COVID-19), whereas among males, the most pronounced gap was in those aged 60–74 years (−10.71% Pre-COVID-19 and −12.07%, During COVID-19).

**Table 5:** Colorectal cancer screening in the social housing and complement cohorts in the “Pre” (2018/2019) and “During” (2020/2021) COVID-19 time periods, stratified by age and sex.

**Sex**	**Age group (years)**	**Eligible population (2018/2019)**					**Eligible population (2020/2021)**				
				**Proportions up-to-date** ***n*** **(%)**					**Proportions up-to-date** ***n*** **(%)**		
		**Complement cohort** ***n***	**Social housing cohort** ***n***	**Complement cohort** ***n*** **(%)**	**Social housing cohort** ***n*** **(%)**	**Difference 95% CI Social housing vs. complement cohort (Reference)** ^*^	**Complement cohort** ***n***	**Social housing cohort** ***n***	**Complement cohort** ***n*** **(%)**	**Social Housing Cohort** ***n*** **(%)**	**Difference 95% CI Social housing vs. complement cohort (Reference)** ^*^
Female	50–59	832,978	20,884	561,237 (67.38)	12,511 (59.91)	−7.47% (−8.14 to −6.80%)	1,014,397	25,495	661,979 (65.26)	14,419 (56.56)	−8.71% (−9.33 to −8.09%)
	60–74	1,184,427	37,295	897,677 (75.79)	26,023 (69.78)	−6.01% (−6.48 to −5.54%)	1,056,506	31,703	767,830 (72.68)	20,754 (65.46)	−7.22% (−7.75 to −6.69%)
	Overall	2,017,405	58,179	1,458,914 (72.31)	38,224 (65.70)^**^	−6.61% (−7.00 to −6.22%)	2,070,903	57,198	1,429,809 (69.04)	34,948 (61.10)^**^	−7.94% (−8.34 to −7.53%)
Male	50–59	817,926	15,953	490,596 (59.98)	8,005 (50.18)	−9.80% (−10.58 to −9.02%)	993,553	18,984	572,539 (57.63)	8,851 (46.62)	−11.01% (−11.73 to −10.29%)
	60–74	1,100,625	25,665	799,641 (72.65)	15,898 (61.94)	−10.71% (−11.31 to −10.11%)	976,720	21,969	680,346 (69.66)	12,652 (57.59)	−12.07% (−12.73 to −11.41%)
	Overall	1,918,551	41,618	1,290,237 (67.25)	23,692 (56.93)^**^	−10.32% (−10.80 to −9.84%)	1,970,273	40,953	1,252,885 (63.58)	21,320 (52.06)^**^	−11.52% (−12.01 to −11.03%)
Overall	50–59 (sex standardized)	1,650,904	36,837	1,051,833 (63.71)	20,293 (55.09)	−8.62% (−9.14 to −8.11%)	2,007,950	44,479	1,234,518 (61.48)	22,970 (51.64)	−9.84% (−10.31 to −9.37%)
	60–74 (sex standardized)	2,285,052	62,960	1,697,318 (74.28)	41,556 (66.00)	−8.28% (−8.65 to −7.91%)	2,033,226	53,672	1,448,176 (71.23)	33,106 (61.68)	−9.55% (−9.96 to −9.13%)
	Overall (age and sex standardized)	3,935,956	99,797	2,749,151 (69.84)	61,300 (61.42)	−7.42% (−7.72 to −7.12%)	4,041,176	98,151	2,682,694 (66.38)	55,645 (56.69)	−9.69% (−10.00 to −9.38%)

^*^A negative sign implies a lower proportion of individuals in social housing compared to the complement cohort.

^**^Age- and sex-standardized rates for social housing using the complement cohort as reference.

## Discussion

This study demonstrates the widening disparities in the uptake of preventive health services among social housing residents compared to the complement cohort during the COVID-19 pandemic. Across age groups, older adults exhibited higher influenza vaccination and cancer screening rates, except for cervical cancer screening.

### Influenza vaccination

During the COVID-19 pandemic, influenza vaccine uptake patterns shifted across populations, revealing growing disparities between individuals living in social housing and those in the complement cohort. While public health campaigns initially promoted both influenza and COVID-19 vaccinations, our findings emphasize that compared to the general population, residents of social housing were less likely to receive the influenza vaccine during the pandemic, potentially due to reduced access to in-person health services, competing health priorities (e.g., prioritizing COVID-19 vaccinations), and a lack of targeted outreach (23–25). The transient nature of COVID-19 focused vaccine campaigns may have further widened existing disparities, reinforcing the need for sustained, community-based interventions to support influenza vaccination in marginalized populations (26). This differs from the literature which demonstrates that influenza vaccination rates did not change significantly before, during, and after the pandemic in retirement residences or older adults-care centres (23). Among adults aged 18–64 without chronic medical conditions, the decline in vaccination was most pronounced during the pandemic, with an odds ratio of 0.82 (95% CI: 0.69–0.98) in the 2021–2022 season compared to 2018–2019 (23).

### Breast cancer screening (mammography)

In Ontario, organized breast cancer screening services were paused in early 2020, resulting in a substantial backlog. Although services resumed later that year, the recovery in screening participation was uneven (27). The COVID-19 pandemic significantly disrupted breast cancer screening programs across Canada, with a pronounced impact on vulnerable populations, including residents of social housing. Our findings show that the disparities in mammography screenings between social housing residents and the general population were already substantial before the pandemic. The overall mammography screening rates for the social housing cohort were 46.37% before the pandemic, dropping to 17.79% during the pandemic. While this appears to be comparable to general population's drop in mammography screening from 56.95 to 21.87%, the narrowing gap is likely due to a “floor-effect” since the pre-pandemic screening rate among social housing residents was already low. Fu et al. (18) reported a 93.4% decrease in weekly bilateral screening mammography rates among average-risk women aged 50–74 in Ontario at the onset of the pandemic, followed by a gradual weekly increase of 8.4% until the end of 2020. However, screening rates among women who were immigrants or had limited access to material resources remained low before and during the pandemic, indicating that existing disparities had persisted during the recovery phase (18). Similarly, Lofters et al. (27) found that the pandemic exacerbated pre-existing disparities in breast cancer screening among individuals living in lower-income neighborhoods and for immigrants significantly widened during the pandemic. The proportion of women in the general population up to date on breast cancer screening in Ontario decreased from 61.1% before the pandemic to 51.7%, showing almost a 10% decrease, compared to our study which shows a 35% decrease (27). This may be because our study only covers the time period 2020–2021 whilst the Lofters et al. (27) study captures later data up to May 2022, including post-pandemic recovery. The study emphasized that policy makers should prioritize improving access to team-based primary care for people who are immigrants and/or living with low income to address these screening disparities (27). A population-based cohort study by Agarwal et al. (8) showed that, prior to the pandemic, women aged 50–69 living in social housing in Ontario had mammography screening rates that were 10%−11% lower than non-residents (8). Our findings confirm that this gap persisted during the pandemic, underscoring the need for targeted interventions to improve screening uptake in social housing populations.

### Cervical cancer screening (Pap test)

Cervical cancer screening rates declined in both cohorts during the pandemic, with consistently lower rates among residents of social housing. However, we observed a slightly narrower gap in screening rates between the social housing and general populations. Routine cervical cancer screening programs were suspended or adapted in several provinces during the pandemic (28, 29). In the first 6 months of the COVID-19 pandemic, Ontario experienced a 63.8% decrease in Pap tests compared to the same period in 2019 (30). A pan-Canadian survey further reported that 15.8% of healthcare professionals observed a 75% or greater reduction in Pap tests during the pandemic and 56.9% reported delays in scheduling these tests (31).

### Colorectal cancer screening

Screening for colorectal cancer is itself a challenging test, requiring an understanding of the health consequences and overcoming certain barriers in order to actually have the test, and low socioeconomic status is associated with poorer uptake (32, 33). Therefore, it is not surprising that residents of social housing, who often face intersecting barriers such as lower health literacy, limited access to primary care, and socioeconomic instability experienced disproportionate declines in colorectal cancer screening participation. In contrast, while the general population also experienced disruptions, the rate of decline in screening is disproportionately higher in the social housing cohort. This raises important concerns for post-pandemic recovery efforts, which must ensure that all individuals receive the screenings they missed (19, 34). Individuals in social housing are likely to remain at a disadvantage due to structural and systematic barriers, whereas individuals in the general population, particularly those with a higher income, may be better positioned to resume or access screening services as they become available. A population-based study in Ontario observed a 3.9% decline in overall colorectal cancer screening from 65.9% in March 2019 to 62.0% in March 2022, with larger declines in low-income neighborhoods (27). Recovery was also slower among low-income populations, raising concerns about delayed diagnoses and poorer long-term health outcomes (6, 19, 27, 35).

Preventive health services remain inequitable for residents of social housing, who often face barriers such as limited access to care, low health literacy, and a lack of tailored outreach (6–8, 11, 36). These disparities worsened during the COVID-19 pandemic and underscore the need for equity-focused policies, community-based delivery models, and robust data systems to monitor outcomes by housing status. Integrating preventive health services into routine community care through mobile clinics and trusted, culturally tailored outreach is essential, given that vaccination and screening data are not consistently tracked by housing status (37, 38). Preventive care should also include embedded navigation services within primary care to support uptake. Given the low cervical cancer screening rates before the pandemic, expanding human papillomavirus self-sampling, increasing access to community-based services, and strengthening infrastructures are necessary during public health emergencies (39, 40). For colorectal cancer screening, recovery strategies such as mailed fecal immunochemical test (FIT) kits and community outreach are critical to engage individuals who have missed screening (41, 42).

### Strengths and limitations

A key strength of this study is the use of large, population-based, linked administrative health datasets from Ontario, which allowed for comprehensive and robust comparisons between individuals living in social housing and the general population. The study captured real-world healthcare utilization across multiple preventive services including influenza vaccination and cancer screening over both pre-pandemic and pandemic periods, enabling a nuanced assessment of trends and disparities. The linkage of multiple high-quality datasets, including those from the OHIP, OBSP, and OCR, provided reliable identification of preventive service uptake and eligibility.

The study also has limitations. This analysis does not include data beyond 2021 and therefore may not reflect full post-pandemic recovery trends in preventive care. Minor inaccuracies may exist in the social housing cohort due to residents' movement in and out of social housing; however, the impact is likely very minimal, as relocation often occurs between buildings. Rigorous methods were employed to ensure accurate identification of the social housing cohort. Our vaccination data are incomplete, as they capture only physician-administered influenza vaccines and not those provided by pharmacists. This may introduce bias if social housing residents were more likely to obtain vaccines from pharmacists. The use of administrative data limits the ability to capture individual-level factors such as social determinants of health, gender, race, and prevalence of mental health conditions which may influence healthcare utilization. Despite these limitations, the study provides important evidence of the inequitable impact of the COVID-19 pandemic on preventive health service use among a socially and economically disadvantaged population.

## Conclusion

This study highlights persistent and worsening disparities in preventive healthcare services among individuals living in social housing compared to the general population in Ontario. Across influenza vaccinations and breast, cervical, and colorectal cancer screenings, residents of social housing consistently demonstrated lower uptake both before and during the COVID-19 pandemic. While some disparities narrowed slightly during the pandemic, this was often due to declines in service uptake among the general population rather than improvements among the social housing cohort. These findings underscore the compounded vulnerability of socially and economically marginalized populations during public health crises. As healthcare systems continue to recover and prepare for future public health challenges, there is an urgent need for equity-focused policies, sustained outreach, and community-based delivery models tailored to social housing populations.

## Data Availability

The datasets presented in this article are not readily available because the dataset from this study is held securely in coded form at ICES. While legal data sharing agreements between ICES and data providers (e.g., healthcare organizations and government) prohibit ICES from making the dataset publicly available, access may be granted to those who meet pre-specified criteria for confidential access, available at www.ices.on.ca/DAS (email: das@ices.on.ca). Requests to access the datasets should be directed to das@ices.on.ca.
